# *EGFR*突变状态对可手术切除的肺腺癌患者复发及生存的预测价值

**DOI:** 10.3779/j.issn.1009-3419.2013.04.02

**Published:** 2013-04-20

**Authors:** 瑜 董, 营 李, 红 彭, 波 金, 艾弥 黄, 皓 白, 慧 熊, 宝惠 韩

**Affiliations:** 1 200030 上海, 上海交通大学附属胸科医院呼吸内科(董瑜, 李营, 金波, 黄艾弥, 白皓, 韩宝惠); 门诊办公室(彭红) Department of Shanghai Chest Hospital, Shanghai Jiao Tong University, Shanghai 200030, China; 2 201499 上海，上海源奇生物医药科技有限公司 Shanghai Yuanqi Biotechnology Company, Shanghai 201499, China

**Keywords:** *EGFR*突变, 肺腺癌, 无疾病生存期, 总生存期, *EGFR* mutation, Lung adenocarcinoma, Disease-free survival, Overall survival

## Abstract

**背景与目的:**

近年来, 肺腺癌在非小细胞肺癌(non-small cell lung cancer, NSCLC)中的比例正越来越高, 对肺腺癌预后的研究有着极其重要的意义。表皮生长因子受体(epidermal growth factor receptor, EGFR)突变状态是否会影响到手术切除的肺腺癌患者的复发及生存, 国内尚缺乏报道。本研究旨在探讨*EGFR*突变状态与可手术肺腺癌的复发及生存的关系。

**方法:**

回顾性分析301例Ⅰa期-Ⅲa期手术切除肺腺癌患者的复发及生存资料。采用荧光定量PCR法筛查+基因测序法确认检测*EGFR*基因突变状态。

**结果:**

*EGFR*突变率为52.5%(158/301)。*EGFR*突变型与野生型的2年无疾病生存期(disease-free survival, DFS)率分别为76.8%、83.0%, 5年总生存期(overall survival, OS)率分别为67.7%、65.7%, 皆无统计学差异(*P*=0.252, *P*=0.715)。进一步的亚组分析显示, Ⅰ期、Ⅱ期患者中突变型与野生型的2年DFS率、5年OS率数值相近且无统计学差异。而Ⅲa期患者中突变型的中位DFS、2年DFS率为12.2个月、21.1%, 明显低于野生型的22.2个月、42.1%, 但无统计学差异(*P*=0.584, *P*=0.295);突变型的中位OS、5年OS率为34.0个月、30.8%, 野生型为38.7个月、22.9%, 无统计学差异(*P*=0.907, *P*=0.444)。

**结论:**

在可手术肺腺癌患者中, 仅Ⅲa期患者*EGFR*突变型较野生型有复发早的趋势, 但无统计学差异。*EGFR*突变状态与术后复发及长期生存无关。

## Abstract (en)

**Background and objective:**

At present the proportion of lung adenocarcinomas in NSCLC is higher than before.Thus, the study on prognosis of lung adenocarcinoma is extremely important.The predictive value of epidermal growth factor receptor (EGFR) mutations for prognosis in patients with resected lung adenocarcinomas has not be reported in China.The aim of this study is to analyze the association between the *EGFR* mutations and the outcomes of patients with resected lung adenocarcinomas.

**Methods:**

Total of 301 patients with stage Ⅰa-Ⅲa resected lung adenocarcinomas whose survival had been identified were retrospectively analyzed.Their *EGFR* mutations were detected by real-time quantitative PCR and DNA sequencing technology together.

**Results:**

The proportion of *EGFR* mutation was 52.5% (158/301).The 2-year (disease-free survival, DFS) of *EGFR*-mutant group and wild-type *EGFR* group was 76.8%, 83.0%;5-year overall survival (OS) of them was 67.7%, 65.7%.There was no significant difference for two groups (*P*=0.252, *P*=0.715).Further analysis for subgroups shows that the 2-year DFS, 5-year OS of mutant group and wild-type group in stage Ⅰ-Ⅱ was similar.For the patients with stage Ⅲa, the median DFS, 2-year DFS in mutant group was 12.2 months, 21.1%, lower than the 22.2 months, 42.1% in wild-type group.But there was no significant difference for both groups (*P*=0.584, *P*=0.295).The median OS, 5-year OS was 34.0 months, 30.8% in mutant group, while 38.7 months, 22.9% in wild-type group.There was no significant difference for both groups (*P*=0.907, *P*=0.444).

**Conclusion:**

*EGFR*-mutant group with resected lung adenocarcinomas has a tendency of lower DFS than the wild-type *EGFR* group only in stage Ⅲa, but there was no significant difference for both groups.The *EGFR* mutation status was not associated with disease-free survival or overall survival in resected lung adenocarcinomas.

中国的流行病学调查显示, 肺癌在各种肿瘤中的发生率及死亡率均占据首位^[1]^。绝大多数肺癌都是非小细胞肺癌, 能够手术的非小细胞肺癌相对预后较好, 然而约30%的早期肺癌仍在5年内死亡。

近年来, 在非小细胞肺癌中, 肺腺癌的发生率有越来越高的趋势, 对肺腺癌预后的研究有着极其重要的意义。研究^[2]^表明, 各种肺腺癌的驱动基因在肿瘤的发生发展中起着举足轻重的作用, 其中表皮生长因子受体(epidermal growth factor receptor, EGFR)最早受到广泛关注, EGFR酪氨酸激酶抑制剂(tyrosine kinase inhibitors, TKIs)的应用是肺癌治疗中历史性的突破。部分研究^[2-4]^显示有*EGFR*突变的晚期肺腺癌患者对化疗的有效率较野生型患者高。而*EGFR*突变状态对于手术切除的肺腺癌患者的预后作用则颇有争议^[5-10]^, 目前尚缺乏针对中国人群的大样本研究。本研究旨在探讨*EGFR*突变状态与可手术切除的肺腺癌患者术后复发及长期生存的关系。

## 1 材料与方法

### 1.1 患者资料

对2004年-2006年行手术切除的301例亚裔、*EGFR*突变状态明确的Ⅰa期-Ⅲa期肺腺癌患者进行回顾性分析。所有患者的临床基本信息及术后总生存期资料完整, 218例患者的术后无疾病生存期资料完整。121例Ⅱa期-Ⅲa期患者接受了规范的术后辅助化疗, 180例Ⅰa期-Ⅰb期患者未接受术后辅助化疗。

### 1.2 *EGFR*突变检测

检测标本为手术切除标本组织, *EGFR*突变检测采用荧光定量PCR法筛查, 阳性标本采用基因测序法再次确认。301例手术标本皆进行了*EGFR*基因的外显子18、19、20、21的检测。

### 1.3 数据统计

分期按照肺癌TNM分期(第7版)进行。*EGFR*突变定义为外显子19缺失突变和/或外显子21置换突变和/或外显子18突变, 包括这3种外显子的双阳性突变。目前的研究已证实EGFR外显子20突变为耐药突变^[11]^, 故本研究将未检测到突变及外显子20突变者都归为*EGFR*野生型。无疾病生存期(disease free survival, DFS)定义为自手术至出现肿瘤复发的时间。总生存期(overall survival, OS)定义为自手术至死亡的时间。无进展生存期(progression free survival, PFS)定义为从治疗到出现肿瘤进展的时间。

### 1.4 统计学分析

采用SPSS 16.0软件进行统计分析。率的比较采用卡方检验。采用*Kaplan-Meier*法绘制生存曲线并进行*Log-rank*检验。采用*Cox*多因素分析评价术后无疾病生存期及术后总生存期与基线特征之间的关系。*P* < 0.05为差异具有统计学意义。

## 2 结果

### 2.1 患者资料

301例手术切除的肺腺癌患者中, *EGFR*总突变率为52.5%(158/301);其中单纯外显子19突变最高(49.4%, 78/158)、单纯外显子21突变其次(33.5%, 53/158)、部分标本为双阳性突变(13.3%, 21/158)、单纯外显子18突变最低(3.8%, 6/158)。女性*EGFR*突变率(58.0%, 91/157)高于男性(46.5%, 67/144)(*P*=0.047);不吸烟或吸烟 < 400年支者(59.6%, 130/218)高于吸烟≥400年支者(33.7%, 28/83)(P < 0.001);年龄 > 60岁者(57.4%, 74/129)高于≤60岁者(48.8%, 84/172)(*P*=0.143);301例患者中, Ⅰa期、Ⅰb期180例(59.8%)、Ⅱa期、Ⅱb期47例(15.6%)、Ⅲa期74例(24.6%), 各组分期的*EGFR*突变率无差异(*P*=0.992)(表 1)。所有患者的末次随访时间为2012年12月, 中位随访时间为79.1个月。121例Ⅱa期、Ⅲa期患者均接受了规范的术后辅助化疗, 180例Ⅰa期-Ⅰb期患者未接受术后辅助化疗。

**1:** 301例手术切除肺腺癌患者一般资料 Characteristics in patients with resected lung adenocarcinomas

Characteristics	*EGFR* (+)	*EGFR* (-)	*P*
No of patients	158 (52.5%)	143 (47.5%)	
Subtype of EGFR		-	
Exon 19	78 (49.4%)	-	
Exon 21	53 (33.5%)	-	
Exon 18	6 (3.8%)	-	
Double positive	21 (13.3%)	-	
Gender			0.047
Female	91 (58.0%)	66 (42.0%)	
Male	67 (46.5%)	77 (53.5%)	
Smoking history			< 0.001
Never or mild smoking (< 400 cigarette years)	130 (59.6%)	88 (40.4%)	
Heavy smoking (≥400 cigarette years)	28 (33.7%)	55 (66.3%)	
Age (yr)			0.143
> 60	74 (57.4%)	55 (42.6%)	
≤60	84 (48.8%)	88 (51.2%)	
Surgical stages			0.992
Ⅰa, Ⅰb	94 (52.2%)	86 (47.8%)	
Ⅱa, Ⅱb	25 (53.2%)	22 (46.8%)	
Ⅲa	39 (52.7%)	35 (47.3%)	
EGFR:epidermal growth factor receptor.			

### 2.2 *EGFR*突变状态与术后DFS的关系

218例DFS资料完整的患者中, *EGFR*突变型(112/218)与野生型(106/218)皆未达到术后中位DFS, 生存分析显示两组DFS无统计学差异(*P*=0.731)(图 1A)。*EGFR*突变型与野生型组的2年DFS率分别为76.8%(86/112)、83.0%(88/106), 两组相比无统计学差异(*P*=0.252)(表 2)。包括吸烟史、性别、年龄、手术分期、*EGFR*突变状态的多因素分析显示, 性别(*P*=0.016)、年龄≤60岁(*P*=0.039)、手术分期(*P* < 0.001)为独立的术后DFS影响因子; 男性、年龄≤60岁、分期较晚者复发早。

**1:**
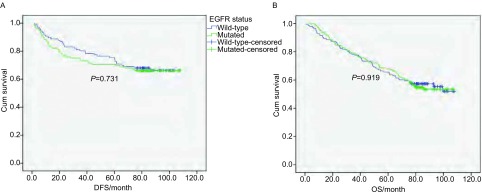
*EGFR*突变状态与术后DFS及OS的关系。A:218例患者*EGFR*突变状态与术后DFS的生存分析, 其中突变型患者112例, 野生型患者106例; B:301例患者*EGFR*突变状态与术后OS的生存分析, 其中突变型患者158例, 野生型患者143例。 DFS and OS for*EGFR* mutated and wild-type patients.A:DFS in 218 patients, mutated *EGFR*:*n*=112, wild-type *EGFR*:*n*=106;B:OS in 301 patients, mutated *EGFR*:*n*=158, wild-type *EGFR*:*n*=143.DFS:Disease-free survival; OS:overall survival; EGFR:epidermal growth factor receptor.

**2:** 不同分期患者的*EGFR*突变状态与2年无疾病生存率、5年生存率的关系 the relationship of EGFR status and 2 year-DFS, 5 year-OS by stages

Surgical stages	2 year-DFS (*n*=218)	*P*	5 year-OS (*n*=301)	*P*	EGFR-TKIs (*n*)
All stages					
*EGFR* (+)	76.8% (86/112)	0.252	67.7% (107/158)	0.715	13
*EGFR* (-)	83.0% (88/106)		65.7% (94/143)		9
Ⅰa, Ⅰb					
*EGFR* (+)	89.9% (71/79)	0.311	83.0% (78/94)	0.728	3
*EGFR* (-)	94.4% (67/71)		84.9% (73/86)		5
Ⅱa, Ⅱb					
*EGFR* (+)	78.6% (11/14)	0.999	68.0% (17/25)	0.526	2
*EGFR* (-)	81.3% (13/16)		59.1% (13/22)		1
Ⅲa					
*EGFR* (+)	21.1% (4/19)	0.295	30.8% (12/39)	0.444	8
EGFR (-)	42.1% (8/19)		22.9% (8/35)		3
EGFR-TKIs:EGFR tyrosine kinase inhibitors.					

### 2.3 *EGFR*突变状态与术后OS的关系

301例患者中, *EGFR*突变型(158/301)与野生型(143/301)皆未达到术后中位OS, 生存分析显示两组OS无统计学差异(*P*=0.919)(图 1B)。*EGFR*突变型与野生型组的5年OS率分别为67.7%(107/158)、65.7%(94/143), 两组相比无统计学差异(*P*=0.715)(表 2)。包括吸烟史、性别、年龄、手术分期、*EGFR*突变状态的多因素分析显示, 性别(*P*=0.024)、手术分期(*P* < 0.001)为独立的术后OS影响因子; 男性、分期较晚者预后差。

### 2.4 不同分期亚组中*EGFR*突变状态与术后DFS及OS的关系

#### 2.4.1 Ⅰ期患者的*EGFR*突变状态与术后DFS及OS的关系

将所有患者根据不同分期进行进一步的亚组分析后, 结果显示:Ⅰ期患者中, *EGFR*突变型(79/150)与野生型(71/150)皆未达到术后中位DFS, 两组DFS无统计学差异(*P*=0.519);突变型(94/180)与野生型(86/180)皆未达到术后中位OS, 两组OS无统计学差异(*P*=0.975)(图 2A、图 2B); 突变型的2年DFS率、5年OS率分别为89.9%(71/79)、83.0%(78/94), 与野生型的94.4%(67/71)、84.9%(73/86)相比无统计学差异(*P*=0.311, *P*=0.728)(表 2)。

**2:**
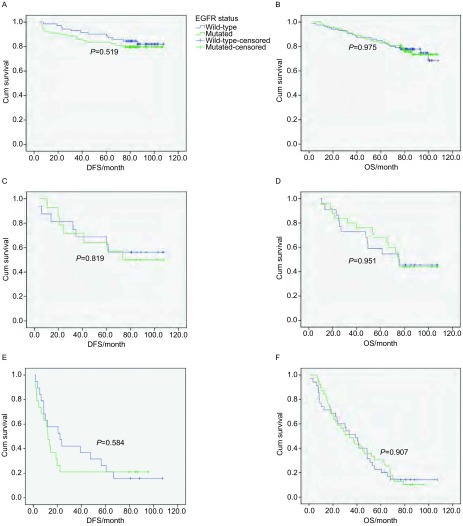
不同分期亚组中*EGFR*突变状态与术后DFS及OS的关系。A:Ⅰ期患者*EGFR*突变状态与术后DFS的生存分析, 其中突变型79例, 野生型71例; B:Ⅰ期患者*EGFR*突变状态与术后OS的生存分析, 其中突变型94例, 野生型86例; C:Ⅱ期患者*EGFR*突变状态与术后DFS的生存分析, 其中突变型14例, 野生型16例; D:Ⅱ期患者*EGFR*突变状态与术后OS的生存分析, 其中突变型25例, 野生型22例; E:Ⅲa期患者*EGFR*突变状态与术后DFS的生存分析, 其中突变型19例, 野生型19例; F:Ⅲa期患者*EGFR*突变状态与术后OS的生存分析, 其中突变型39例, 野生型35例。 DFS and OS by stages.A:DFS in stage Ⅰ, mutated *EGFR*:*n*=79, wild-type *EGFR*:*n*=71;B:OS in stage Ⅰ, mutated *EGFR*:*n*=94, wild-type *EGFR*:*n*=86;C:DFS in stage Ⅱ, mutated *EGFR*:*n*=14, wild-type *EGFR*:*n*=16;D:OS in stage Ⅱ, mutated *EGFR*:*n*=25, wild-type *EGFR*:*n*=22;E:DFS in stage Ⅲa, mutated *EGFR*:*n*=19, wild-type *EGFR*:*n*=19;F:OS in stage Ⅲa, mutated *EGFR*:*n*=39, wild-type *EGFR*:*n*=35.

#### 2.4.2 Ⅱ期患者的*EGFR*突变状态与术后DFS及OS的关系

Ⅰ期患者中, *EGFR*突变型(14/30)术后中位DFS为76.3月, 而野生型(16/30)尚未达到术后中位DFS, 两组DFS无统计学差异(*P*=0.819);突变型(25/47)与野生型(22/47)的术后中位OS分别为75.2个月、75.5个月, 两组OS无统计学差异(*P*=0.951)(图 2C、图 2D); 突变型的2年DFS率、5年OS率分别为78.6%(11/14)、68.0%(17/25), 与野生型的81.3%(13/16)、59.1%(13/22)相比无统计学差异(*P*=0.999, *P*=0.526)(表 2)。

#### 2.4.3 Ⅲa期患者的*EGFR*突变状态与术后DFS及OS的关系

Ⅲa期患者中, *EGFR*突变型(19/38)术后中位DFS为12.2个月, 明显低于野生型(19/38)的22.2个月, 但两组DFS无统计学差异(*P*=0.584);突变型(39/74)与野生型(35/74)术后中位OS分别为34.0个月、38.7个月, 两组OS无统计学差异(*P*=0.907)(图 2E、图 2F); 突变型的2年DFS率21.1%(4/19)明显低于野生型的42.1%(8/19), 但无统计学差异(*P*=0.295);突变型的5年OS率30.8%(12/39)略高于野生型的22.9%(8/35), 但无统计学差异(*P*=0.444)(表 2)。

### 2.5 复发后EGFR-TKIs治疗

共有22例术后复发的患者接受过EGER-TKIs治疗(表 2), 其中*EGFR*突变型患者13例, 中位复发后OS为31.7个月, TKIs治疗的中位PFS为19个月; 野生型患者9例, 中位复发后OS为38.4个月, TKIs治疗的中位PFS为6个月。

## 3 讨论

本研究显示, *EGFR*突变状态对可手术切除的肺腺癌患者术后DFS及OS无预测价值。对不同分期的亚组分析显示, Ⅰa期-Ⅱb期患者中*EGFR*突变型与野生型的2年DFS率、5年OS率无明显差异, 中位DFS、OS尚未达到, 需进一步随访; 而在Ⅲa期患者中, 虽然统计学无差异, 但*EGFR*突变型相对野生型患者似乎DFS更短而OS相仿。

多因素分析显示, 性别、手术分期是长期生存的独立影响因子, 这在先前的研究^[12]^中已经得到验证。而*EGFR*突变状态不是长期生存的独立影响因子, 与既往研究^[5, 6]^一致。

对*EGFR*突变状态与手术切除的肺腺癌患者术后复发及生存的关系尚无一致意见^[5-10]^, 尤其是缺乏针对中国人群的大样本研究。既往的研究^[5, 9, 10]^往往只分析EGFR与OS的关系, 未分析与DFS的关系且未进行不同分期的亚组分析, 而我们的研究发现*EGFR*突变状态对不同分期患者DFS和OS的影响可能是不同的。最近的两项研究^[6, 7]^发现, *EGFR*突变型术后患者较野生型患者更早复发但长期生存无区别, 这与我们的研究结果相符, 可能与其中Ⅲa期比例相对较高有关。

尽管有研究推测晚期肺癌中, *EGFR*突变者对初始治疗的敏感性更高, 表现为更高的近期疗效, 但这种现象仅在晚期患者中得到证实^[2-4, 13, 14]^。而有研究^[5, 15-19]^推测EGFR激活突变使肿瘤细胞增殖速度更快, 使肿瘤细胞更具侵袭性, 更容易发生转移, 一旦进展可能发展更为迅速。在手术切除的患者中, 正如Mak^[4]^的研究结果一样, 肿瘤负荷几乎为0, *EGFR*突变对化疗的敏感性得不到优势体现, 从而无法延长术后复发时间。并由于*EGFR*突变者肿瘤的侵袭性更强, 使得这类患者术后更易复发, 而一旦复发, *EGFR*突变者对化疗或靶向治疗的敏感性就产生优势, 使得相应的复发后生存时间较长, 而这两方面的正负作用相抵, 使得*EGFR*突变者OS与野生型患者相仿。本研究中Ⅲa期患者大部分为N2手术分期, 且可能存在潜在的血道转移, 术后复发早, 加上*EGFR*突变使得肿瘤侵袭性更强, 这可能是导致Ⅲa期*EGFR*突变型的术后患者DFS更短的原因。

研究入组的患者为2004年-2006年手术切除的肺腺癌, 当时靶向治疗药物未在中国广泛应用, 导致本研究中使用TKIs者仅占7.3%(22/301), 这可能也使得部分患者即使*EGFR*突变也不能得到有效的个体化治疗, 从而不能延长生存。而且22例接受TKI治疗的患者中*EGFR*突变者大部分手术分期较晚, 虽然TKIs治疗的中位PFS较长, 但OS仍较相对早期的*EGFR*野生型者短。中国人群的一项研究中^[14]^总体EGFR-TKIs的使用率为47.6%(69/145), 结果显示不管*EGFR*突变状态如何, EGFR-TKIs的应用都可以一定程度的延长患者OS, *EGFR*突变型患者OS优于野生型。本研究结果显示, 在EGFR-TKIs使用率低的状态下, *EGFR*突变型患者的OS与野生型患者相仿, 这说明*EGFR*突变本身不是肺腺癌的预后因素。但根据以往研究, *EGFR*突变是肺癌靶向治疗的预测因素, 若随着EGFR-TKIs更普遍的应用, 将来术后*EGFR*突变型患者的总体生存可能会比野生型患者得到更好的延长。

本研究中Ⅰa期-Ⅱb期患者*EGFR*突变型与野生型都未达到中位DFS、OS, 尚需进一步随访。而Ⅲa期患者中虽然数据显示有差异, 但统计学无差异, 这可能由于样本量较小, 将来需扩大样本量继续研究。
